# Enhanced Interaction between Warfarin and High-Dose Ketoconazole: A Case Report

**DOI:** 10.1155/2009/315687

**Published:** 2009-12-09

**Authors:** Cynthia A. Jackevicius, Mannhu N. Ton

**Affiliations:** ^1^Department of Pharmacy Practice and Administration, College of Pharmacy, Western University of Health Sciences, CA 91766-1854, USA; ^2^VA Greater Los Angeles Healthcare System, West Los Angeles Healthcare Center, CA 90073, USA; ^3^Department of Pharmacy, University Health Network, Toronto, Canada M5G 2C4; ^4^Department of Health Policy, Management and Evaluation, Faculty of Medicine, University of Toronto, Toronto, Canada M5S 1A1; ^5^Institute for Clinical Evaluative Sciences, Toronto, Canada M4N 3M5

## Abstract

This case describes the increased anticoagulation effect associated with the use of high-dose ketoconazole. A 59-year-old man treated with warfarin for aortic valve replacement was prescribed high-dose ketoconazole and hydrocortisone for the treatment of prostate cancer. Despite lowering the warfarin dosage by 35% during the start of high dose ketoconazole, an additional dose reduction was required subsequently when the INR rose from 2.62 to 3.82 within nine days. After a total dose reduction of 43%, the INR returned to therapeutic range within two weeks. The Naranjo probability scale revealed a probable adverse reaction of increased anticoagulant effect associated with high dose ketoconazole. Due to the inhibition of warfarin metabolism by ketoconazole, patients taking high dose ketoconazole concomitantly with warfarin may need their warfarin dosage reduced by more than is currently recommended, as well as receive more frequent INR monitoring to avoid over anticoagulation.

## 1. Introduction

Prostate cancer is the most common male malignancy, ranking third in cancer incidence worldwide and sixth in cancer mortality among men [1]. In advanced prostate cancer, androgen deprivation (AD) is the primary therapeutic approach. Despite initial response rates of 80%–90% to AD therapy, nearly all men experience progression after 18–24 months [2]. Alternatives to AD are secondary hormonal interventions such as high-dose ketoconazole (HDK), aminoglutethimide, or diethylstilbestrol [2, 3]. Ketoconazole is an imidazole derivative antifungal, which also has testosterone lowering effects by decreasing testicular and adrenal production of androgens by inhibiting adrenal and gonadal steroid syntheses. At high-doses, ketoconazole inhibits the cholesterol side chain cleavage, P450c17, C17, 20 lyase, 3*β*-hydroxysteroid dehydrogenase, and P450c11 enzymes required for steroid hormone synthesis [4, 5]. Ketoconazole inhibitory effects on steroid biosynthesis are seen only at high-doses [4, 5]. In addition, ketoconazole has been shown to have direct cell killing action on prostate cancer cells [6]. As a result of these mechanisms, HDK is now being used for men with hormone refractory prostate cancer (HRPC).

As an antifungal, ketoconazole is dosed at 200 mg daily, however, for HRPC therapy; doses of 200–400 mg three times a day are used to produce castrate levels of testosterone within 24 hours [7]. Since ketoconazole is a potent inhibitor of cytochrome P450 isoenzymes in the liver, it can increase concentrations and potentiate the effects of some drugs, such as cyclosporine, phenytoin, oral hypoglycemics, and oral anticoagulants [7]. Other antifungals, including fluconazole, voriconazole, and miconazole, have highly probable reports of drug interactions with warfarin [8].

## 2. Case Presentation

A 59-year-old man had been treated with warfarin with an INR goal of 3.0 for the last five years after receiving a mechanical aortic valve replacement. His past medical history includes diabetes mellitus type 2, hyperlipidemia, hypertension, depression, chronic obstructive pulmonary disease, gastroesophageal reflux disease, osteoarthritis, and prostate cancer. His medications included albuterol and ipratropium inhaler, benztropine, calcium/vitamin D, guaifenesin/dexamethorphan, docusate, furosemide, glyburide, goserelin, hydrocodone/acetaminophen, lisinopril, lorazepam, metoprolol tartrate, nitroglycerin sublingual, omeprazole, potassium chloride, prochlorperazine, quetiapine, simvastatin, terazosin, and trazodone. His INR had been therapeutic in the past two months on a usual weekly dose of 52.5 mg. His only other elevated INR prior to this period was when antibiotics were started for an acute infection, and his INR increased from 3.03 to 4.27.

In March 2006, the patient was prescribed ketoconazole 400 mg three times a day and hydrocortisone 10 mg daily by his urologist for treatment of prostate cancer. As part of the pharmacist-managed anticoagulation program, the pharmacist decreased the warfarin weekly dose by 35% to avoid overanticoagulation. This dose adjustment was based on the recommendation from standard clinical references to reduce the warfarin dose by one-third [9]. The patient returned to the anticoagulation clinic on days 3, 9, and 20 after the initiation of HDK. His INR readings were 2.61, 3.82, and 3.16, respectively. During these three visits, he denied bleeding or dietary and medication changes. Due to the increase in INR on day 9 with no other reasons contributing to the INR elevation, the warfarin dosage was further decreased to 30 mg per week, resulting in a total warfarin dose reduction of 43% since ketoconazole initiation. On day 20, his INR was therapeutic at 3.16 and warfarin control was reestablished within the therapeutic goal thereafter.

## 3. Discussion

In this case, despite lowering the warfarin dosage by 35% during the start of HDK, an additional dose reduction was required when the INR increased to 3.82 within nine days. A total dose reduction of 43% was required to bring the anticoagulant effect to therapeutic goal and to avoid bleeding complications. No new medications besides ketoconazole and hydrocortisone were started during this period of time. The medications listed previously were chronic medications that the patient had been taking for years, except for calcium/vitamin D, guaifenesin/dexamethorphan, and glyburide which were started within two months prior to the initiation of ketoconazole and hydrocortisone. Among the concurrent medications with warfarin, only omeprazole, glyburide, and ketoconazole show an enhanced anticoagulation effect [9]. The drug interactions between omeprazole and warfarin as well as glyburide and warfarin had already been accounted for prior to the start of HDK, thus implicating ketoconazole in the potentiation of the warfarin effect. The Naranjo, the modified Naranjo adverse drug reaction probability scales, and the Holbrook criteria all revealed a probable adverse reaction of an increased anticoagulant effect associated with HDK [8, 10, 11]. 

To our knowledge, this is the first report of the potentiation of oral anticoagulants by HDK. The mechanism of the drug interaction is most likely due to the inhibition of warfarin metabolism. Ketoconazole is a potent inhibitor of cytochrome P450 1A2 and 3A4, with moderate inhibitory effect on isoenzymes 2A6, 2C9, 2C19, and 2D6 [12, 13]. As warfarin is a substrate for several isoenzymes (CYP 1A2, 2C9, 2C19, 3A4) that are inhibited by ketoconazole, inhibition of these isoenzymes may enhance the warfarin anticoagulant effect [13].

While clinicians may readily acknowledge the interaction between ketoconazole and warfarin, supporting data are sparse. In the widely cited evidence-based overview of warfarin drug interactions, Holbrook and colleagues indicate that ketoconazole has no effect on warfarin at the probable level, based on a pharmacokinetic study assessing two patients receiving concurrent warfarin and ketoconazole [8, 14]. Only one case report has been published demonstrating warfarin potentiation with low-dose ketoconazole (200 mg/day). This report suggested more frequent INR monitoring and reduction of the warfarin dosage by one-third [15]. Current drug interaction references cite this one report to guide dosage adjustment for this interaction. We provide the second case report and show that with the use of HDK, a dose adjustment of one-third may not be adequate. In our case, a total dose reduction of 43% was required to bring the INR to therapeutic levels. In light of our case, for patients taking high-dose ketoconazole concomitantly with warfarin, it may be prudent to consider a warfarin dosage reduction of more than 35%, in conjunction with more frequent INR monitoring.

## 4. Conclusion

The use of HDK therapy for hormone refractory prostate cancer has increased. Clinicians must be cognizant and use caution when HDK is used with warfarin as it can greatly potentiate the anticoagulation effect, leading to an increased risk of bleeding. This drug-drug interaction may be minimized by more frequent INR monitoring and considering reducing the warfarin dosage by more than the currently recommended 35% in order to achieve the therapeutic goal and to avoid overanticoagulation.
